# Munchausen syndrome and Munchausen syndrome by proxy: a narrative review

**DOI:** 10.1590/S1679-45082017MD3746

**Published:** 2017

**Authors:** Daniel de Sousa, Elton Yoji Kanomata, Ricardo Jonathan Feldman, Alfredo Maluf

**Affiliations:** 1 Hospital Israelita Albert Einstein, São Paulo, SP, Brazil

**Keywords:** Munchausen syndrome, Munchausen syndrome by proxy, Child abuse, Factitious disorders, Síndrome de Munchausen, Síndrome de Munchausen causada por terceiros, Maus-tratos infantis, Transtornos autoinduzidos

## Abstract

The Munchausen syndrome and Munchausen syndrome by proxy are factitious disorders characterized by fabrication or induction of signs or symptoms of a disease, as well as alteration of laboratory tests. People with this syndrome pretend that they are sick and tend to seek treatment, without secondary gains, at different care facilities. Both syndromes are well-recognized conditions described in the literature since 1951. They are frequently observed by health teams in clinics, hospital wards and emergency rooms. We performed a narrative, nonsystematic review of the literature, including case reports, case series, and review articles indexed in MEDLINE/PubMed from 1951 to 2015. Each study was reviewed by two psychiatry specialists, who selected, by consensus, the studies to be included in the review. Although Munchausen syndrome was first described more than 60 years ago, most of studies in the literature about it are case reports and literature reviews. Literature lacks more consistent studies about this syndrome epidemiology, therapeutic management and prognosis. Undoubtedly, these conditions generate high costs and unnecessary procedures in health care facilities, and their underdiagnose might be for lack of health professional's knowledge about them, and to the high incidence of countertransference to these patients and to others, who are exposed to high morbidity and mortality, is due to symptoms imposed on self or on others.

## RESUMO

A síndrome de Munchausen e a síndrome de Munchausen por procuração são condições caracterizadas pela invenção ou pela produção intencional de sinais ou sintomas de doenças, bem como alterações de exames laboratoriais. Indivíduos com esta síndrome fingem que estão doentes e tendem a procurar tratamento, sem ganho secundário, em diferentes serviços de saúde. Ambas as síndromes são condições bem descritas na literatura desde 1951. Elas são frequentemente observadas pelas equipes de saúde em clínicas, enfermarias hospitalares e prontos-socorros. Conduziu-se revisão narrativa, não sistemática da literatura, incluindo relatos de caso, séries de relatos de caso, artigos de revisão indexados no MEDLINE/PubMed de 1951 a 2015. Cada estudo foi revisado por dois especialistas em psiquiatria que, por meio de consenso, escolheram quais estudos seriam incluídos nesta revisão. Apesar da síndrome de Munchausen ter sido descrita pela primeira vez há mais de 60 anos, a maioria dos estudos conduzidos sobre esta condição são relatos de caso e revisões da literatura. A literatura é carente de estudos mais consistentes sobre epidemiologia, manejo terapêutico e prognóstico da síndrome. Sem dúvida, tais condições geram altos custos e procedimentos desnecessários nos serviços de saúde. Seu subdiagnóstico pode se dar pela falta de conhecimento das síndromes por parte dos profissionais de saúde, e à alta incidência de contratransferência aos pacientes e a outros que são expostos à alta morbidade e à mortalidade é justificada pelos sintomas impostos em si mesmo ou em terceiros.

## INTRODUCTION

The term “Munchausen syndrome” was first described in 1951 by Asher^(^ ^1^ ^)^ to characterize individuals who intentionally produce signs and symptoms of a disease and who tend to seek medical or hospital care. Later, in 1977, Meadow used the term “Munchausen syndrome by proxy” to describe children whose mothers produce histories of illness to their children and who support such histories by fabricated physical signs and symptoms, or even by alter laboratory tests.^(^ ^2^ ^)^

The term “Munchausen” is associated with Baron Münchhausen (Karl Friedrich Hieronymus Freiherr von Münchhausen, 1720-1797), to whom fantastic and unreal stories about his life and experiences were attributed.^(^ ^2^ ^)^

Our study reviews the literature about Munchausen syndrome and Munchausen syndrome by proxy. This is a narrative, non-systematic review including case reports, series of case reports, and reviews indexed in PubMed from the first paper published on this subject in 1951 to November 2015. We used the following keywords “Munchausen syndrome”, “Munchausen syndrome by proxy” and “factitious disorders”.

Each study was reviewed by two psychiatric specialists who later, in consensus, selected relevant studies to be included in the review, considering clinical, epidemiogical and treatment-related aspects of these syndromes. In addition, other relevant studies based on judges’ experience by the specialists were included in our review, *i.e.,* state of the art papers about those conditions.

This review seeks to provide basic information on both syndromes for students and health professionals (mainly non-specialists) interested in a general panel of these conditions generally unknown or misdiagnosed, however, seen in emergency rooms, clinical or surgical units or other health care settings. Munchausen syndrome and its variant forms are challenges faced in clinical and surgical practice. For this reason, to briefly review these conditions is important and justified in order to further understand these frequently unrecognized affections.

## CLASSIFICATION AND CLINICAL PRESENTATION

Currently, despite the disseminated use of the term “Munchausen syndrome,” no nosological entity for these two syndromes is described in the International Classification of Diseases. Munchausen syndrome was included in the tenth edition of the International Classification of Diseases^(^ ^3^ ^)^ and classified as intentional production or feigning of symptoms or disabilities either physical or psychological (factitious disorder). Munchausen syndrome by proxy is classified in the category T74.8, *i.e.,* abuse of children, although this term is also used to refer to elderly or disabled person and/or dependent adults who signs or physical symptoms are created by a caregiver and whose laboratory tests were altered.^(^ ^4^ ^)^

Munchausen syndrome has also been called “hospital addiction”, “polysurgical addiction,” and “professional patient syndrome”.^(^ ^3^ ^)^

The Diagnostic and Statistical Manual of Mental Disorders, fifth edition (DSM-5), defines factitious disorders as those imposed on self and on other (previously called “factitious disorders by proxy”).^(^ ^5^ ^)^

Main characteristics of factitious disorder imposed on self are feigning of physical and/or psychological signs and symptoms and induction of injury or disease associated with identified fraud. The diagnosis criteria in DSM-5 to factitious disorder imposed on self are described in chart 1, and the criteria for factitious disorder imposed on other are described in chart 2.^(^ ^5^ ^)^

**Chart 1:** Diagnostic criteria for factitious disorder imposed on self

A. The patient feigns psychological and physical signs and symptoms, or induction of lesion or disease; factitious disorder	
B. The individual presents him/herself to others as ill, impaired or injured	
C. Fraudulent behavior is evident even in the absence of obvious external rewards	
D. Individual's behavior is no longer well explained by a disorder, such as delirium or other psychotic condition	
Specify:	
	Single episode
	Recurrent episodes (two or more events of feigning diseases and/or induction of injury)

Source: American Psychiatric Association (APA). Manual diagnóstico e estatístico e transtornos mentais DMS-5. 5a ed. Tradução de Maria lnês Corrêa Nascimento, Paulo Henrique Machado, Regina Machado Garcez, Rêgis Pizzato, Sandra Maria Mallmann da Rosa. Porto Alegre: Artmed; 2013. Transtorno factício; p. 325-7.^(^^5^^)^

**Chart 2:** Diagnostic criteria for factitious disorder imposed on other (previously named "factitious disorder by proxy”)*

A. Psychological and physical signs and symptoms, or induction lesion or disease on other are feigned in association with identified fraud	
B. Individual presents the other (victim) as ill, impaired or injured	
C. Fraudulent behavior is evident even with absence of obvious external rewards.	
D. Individual's behavior is no longer well explained by a disorder, such as delirium or other psychotic condition	
	Single episode
	Recurrent episodes (two or more events of feigning a disease and/or induction of an injury)

* The agent, not the victim, receives the diagnosis.

Source: American Psychiatric Association (APA). Manual diagnóstico e estatístico e transtornos mentais DMS-5. 5a ed. Tradução de Maria lnês Corrêa Nascimento, Paulo Henrique Machado, Regina Machado Garcez, Rêgis Pizzato, Sandra Maria Mallmann da Rosa. Porto Alegre: Artmed; 2013. Transtorno factício; p. 325-7.^(^^5^^)^

In general, individuals with factitious disorder report their story dramatically, but they are quite vague and inconsistent when asked to provide further details. These patients frequently have a history of pathological lies about any aspect of their history or symptoms (*i.e*., pseudologia fantastica), and they can even have extensive knowledge on medical terminology, routines, and hospital protocols. Students or health professionals have been described, and there is a question of whether the increased incidence of Munchausen syndrome among this population.^(^ ^6^ ^)^

Reports of nonspecific pain and other nonspecific symptoms and request for analgesics are also quite common. If, after extensive investigation complaints, no clear evidence exist that the individual is facing a true clinical condition, such patients may report other somatic and/or psychological problems and produce other nonspecific signs and/or symptoms. Individuals with this disorder can be submitted to multiple unnecessary procedures, including frequent and invasive surgery, without achieving an accurate diagnosis or successful therapy.

In the hospital environment, many of these patients receive few or no visits during hospitalization. Eventually, the fraudulent nature of these signs and symptoms is revealed (*e.g*., the patient is recognized by some professional who previously assisted him/her, or other hospital confirm previous admissions of the patient due to the same health problem and who diagnosis was factitious disorder). However, when these individuals with this disorder are informed that evidences show that symptoms are fraudulent and confronted, they often deny or leave the hospital without formal discharge. Commonly, later on, they end up seek another hospital or health service to be admitted.^(^ ^7^ ^)^

In factitious disorder with predominant psychological signs and symptoms, as shown in chart 3, patients can provide approximated answers to simple questions (*e.g*., 8 times 8 equals 65). The individual can discretely use psychoactive substances to produce symptoms that suggest mental disorder (*e.g*., stimulators to produce uneasiness or insomnia, hallucinogens to alter perception, analgesics to induce euphoria and hypnotics to cause lethargy). The combinations of psychoactive substances can produce extremely uncommon clinical pictures.^(^ ^8^ ^)^

**Chart 3:** Indications of factitious disorder with psychological conditions

Worsening of symptoms after hospital discharge
Symptoms not consistent with those found in a syndrome
Consistent response to treatment
Reports about physical and emotional trauma, but no one can confirm them
Pseudologia fantastica (pathological liar)
Intense relationship with other patients and health care team
Symptoms similar to other patients that appear during hospitalization

Source: American Psychiatric Association (APA). Manual diagnóstico e estatístico e transtornos mentais DMS-5. 5a ed. Tradução de Maria lnês Corrêa Nascimento, Paulo Henrique Machado, Regina Machado Garcez, Rêgis Pizzato, Sandra Maria Mallmann da Rosa. Porto Alegre: Artmed; 2013. Transtorno factício; p. 325-7.^(^^5^^)^

Individuals with factitious disorder with predominant physical sign and symptoms can also be seen as substance abusers, particularly of analgesics and prescribed sedatives. Multiple hospitalizations often lead to iatrogenic general medical conditions (*e.g*., multiple scars because of unnecessary surgeries or adverse drug reactions). Individuals with the chronic form of this disorder can have a “gridiron abdomen” caused by multiple surgical scars. In general, individuals with factitious disorder have difficulty to maintain their job, create family ties, and stable interpersonal relationships. The most common factitious disorders in medical and surgical clinic are shown in chart 4.

**Chart 4:** Most common factitious disorders in medical and surgical clinic

Abdominal pain or recurrent pain in multiple sites^(^ ^9^ ^)^
Unexplainable metabolic and hydroelectrolytic disorders^(^ ^10^ ^–^ ^12^ ^)^
Hard-to-heal wounds and pathological bleeding in different sites^(^ ^13^ ^–^ ^16^ ^)^
Unexplained bleeding^(^ ^13^ ^–^ ^16^ ^)^
Repetitive urinary tract infections, hematuria and proteinuria^(^ ^17^ ^)^
Repetitive infections in different sites^(^ ^18^ ^)^
Genital injuries^(^ ^19^ ^)^
Convulsions^(^ ^20^ ^)^
Skin injuries and repetitive ocular conditions^(^ ^21^ ^,^ ^22^ ^)^
Subcutaneous emphysema^(^ ^23^ ^)^
No accidental poisoning in children, elderly patients or disabled persons^(^ ^24^ ^)^

Source: American Psychiatric Association (APA). Manual diagnóstico e estatístico e transtornos mentais DMS-5. 5a ed. Tradução de Maria lnês Corrêa Nascimento, Paulo Henrique Machado, Regina Machado Garcez, Rêgis Pizzato, Sandra Maria Mallmann da Rosa. Porto Alegre: Artmed; 2013. Transtorno factício; p. 325-7.^(^^5^^)^

Possible predisposing factors for factitious disorder can include presence of other mental disorders or medical conditions in childhood or adolescence that lead to long-term treatments and hospitalizations, resentment against medical professionals, experience in a position related to health area, presence of personality disorders, and important relationship with a physician in the past.^(^ ^8^ ^)^

## PREVALENCE AND COURSE OF THE DISORDER

Information is limited about prevalence of factitious disorders, including Munchausen syndrome. However, its diagnosis is rarely reported and underdiagnosis is evident. The disorder is reported more often among men than women. The course of factitious disorders can be limited to one or more brief episodes, but generally, it is chronic. The disorder normally occurs in the first year of adulthood, and in most of cases after hospitalization of a general medical condition or other mental disorder.^(^ ^8^ ^)^

## DIFFERENTIAL DIAGNOSIS

Factitious disorder diagnosis must be differentiated from general real medical conditions and evident mental disorder. Suspicion for possible mental disorders or general medical conditions that represent a factitious disorder must appear when any combination of the following factors is seen in a hospitalized patient: atypical presentation that is not classified as a general medical condition or an identified mental disorder, symptoms or behaviors present only when the individual is being observed, pseudologia fantastica, atypical behavior at hospital wards (*e.g*., disobedience to hospital rules and excessive arguing with health professionals responsible for the care), unusual grasping of medical terminology and hospital routines, hidden use of substances, evidence of multiple treatments (*e.g*., multiple surgeries, repetitive courses of electroconvulsive therapy), a history of extensive travels, few or no visits during hospitalization, fluctuating clinical course, and rapid development of “complications” or new “disease” in patients who initial investigation was negative. In somatoform disorders, physical complaints that are not plentiful attributed to true general medical condition, but symptoms are not intentional produced. Simulation differs from factitious disorder in that motivation for production of symptoms in simulation is characterized by external incentive whereas in factitious disorder there are no external incentives. Individuals with simulation can seek hospitalization by fabricating symptoms with the aim of obtaining financial compensation, evading the police or simply “a place to spend the night”. However, in most cases, symptoms can “disappear” when they are not useful anymore.^(^ ^7^ ^–^ ^9^ ^)^

## SIMULATION

Simulation is not considered a mental disorder. It is included in the DSM-5 and defined as intentional production of false or exaggerated physical or psychological symptoms motivated by external incentives in order to avoid mandatory military service, avoid work, obtain financial compensation, escape from criminal process, or obtain drugs. Simulation differs from factitious disorder in terms of motivation for production of symptoms. In simulation the incentive is external while factitious disorders lack this incentive.

Simulation is also classified in DSM-5 in the category “Other focus of clinical attention”, but in revised version of the International Classification of Diseases 10^th^, it is classified in Z76.0 – Simulation (conscious) in the item “Persons encountering health services in other circumstances”.^(^ ^5^ ^,^ ^25^ ^)^

According to DSM-5, chart 5 shows elements that strengthen the suspicion of simulation in any combination, although it is not considered a formal diagnosis criterion.

**Chart 5:** Warning signs of simulation

Legal-medicine context of presentation (e.g., the individual is referred to general practitioner by a lawyer for exam, or he does it by himself/herself whereas legal processes and accusations are judged
Important difference between reported stress and incapability of the individual, findings and objective observations
Lack of cooperation during diagnostic evaluation and adherence to treatment regimen prescribed
Presence of antisocial personality disorder.

Source: American Psychiatric Association (APA). Manual diagnóstico e estatístico e transtornos mentais DMS-5. 5a ed. Tradução de Maria lnês Corrêa Nascimento, Paulo Henrique Machado, Regina Machado Garcez, Rêgis Pizzato, Sandra Maria Mallmann da Rosa. Porto Alegre: Artmed; 2013. Transtorno factício; p. 325-7.^(^^5^^)^

## TREATMENT

In general, treatment for factitious disorder is not based on controlled and randomized studies. In 2008 a systematic review on factitious disorders, which included 32 case reports and 13 case series, showed insufficient evidence to evaluate the effectiveness of any management technique for factitious disorders, including psychotherapy, drug treatment, behavioral therapy and multidisciplinary techniques.^(^ ^26^ ^)^ So far, no biologic or psychological therapy has shown efficacy based on reviews and empiric reports of clinicians with experience on this field. No comparative analyses have been carried out between different types of therapeutic approach, although a number of techniques have been described, such as psychodynamic and behavioral techniques.

Some authors stated that involuntary psychiatric hospitalization has been used for patients who put himself/herself at risk and who cannot be treated on an outpatient unit. Such approach is need because most patients, although willing to assume the position of an ill person or put others in this position, do not recognize themselves as having mental disorder, they often do not adhere to treatment, and sometimes run away from their hometown to try to be admitted in another health service by reporting previous clinical features he/she had produced intentionally. Treatment of these patients is extremely difficult; presents very low rates of adherence, poor prognosis; and few cases have improvements. It should be emphasized that most of the treatments reported in case studies or literature reviews were conducted in hospital settings, with few weeks or months of treatment, which could be an important bias in these studies.^(^ ^7^ ^–^ ^9^ ^)^

## DISCUSSION

Munchausen syndrome and Munchausen syndrome by proxy have been described in the medical literature since 1951. These are well-established terms to describe subtypes of factitious disorders.^(^ ^1^ ^)^

Although Munchausen syndrome has been described for more than 60 years, it is clear the scarcity of consistent studies about its epidemiology, therapeutic management and prognosis. Most studies on factitious disorders are case reports and non-systematic literature reviews. So far few systematic and consistent studies that have sought to investigate basic issues, such as epidemiology or even clinical or psychological management of factitious disorders. This lack of trustworthy epidemiologic studies related to these disorders is attributed to the fact that patients, when diagnosed, often do not accept their diagnosis and refuse to adhere to any treatment, and they generally continue to seek other hospitals and health services. The fact that patients frequently do not adhere to and do not cooperate with the treatment or even deny that they have a psychiatric disorder rather than a clinical pathology constitutes a factor that undoubtedly makes it difficult to conduct epidemiological studies or clinical trials to establish therapeutic modalities such as psychotherapy or pharmacotherapy. This fact is an important limitation both to our study and studies included in our review.

Munchausen syndrome and Munchausen syndrome by proxy are associated with high morbidity, and some reports on mortality have been published. Of note is that patients with this syndrome consume resources and time of health care teams because of the unnecessary evaluations and procedures. These disorders can be associated with iatrogenesis if factitious disorder is not detected by the physicians or even by health interdisciplinary team in the hospital or outpatient unit. In addition, when patients with factitious disorders are recognized (which occurs frequently), they are rejected by health professionals that countertransference situations are seen; the health professionals discuss these situations in interdisciplinary teams or even during psychiatric or psychological consultations.^(^ ^10^ ^,^ ^27^ ^)^

The general hospital setting in which patients are hospitalized and assisted by an interdisciplinary team with intensive care, theoretically, is the ideal environment to identify the disorder, start management and take adequate measures, especially in the case of abuse of a child, a senior or disabled person. The adequate diagnosis, management and referral of patients with factitious disorder upon hospital admission is fundamental for good prognosis and reduction of injuries and/or severe adverse events during hospitalization of these individuals.

## CONCLUSION

Munchausen syndrome and Munchausen syndrome by proxy are often not identified and diagnosed by physicians and other health professionals. The lack of identification may lead to many unnecessary laboratory tests and procedures which may prolong hospitalizations and increase costs of health systems. So far, no effective treatments have been demonstrated through well-conducted studies, and no diagnostic criteria exist; these facts may explain the little knowledge of students and health practitioners about these conditions. Munchausen syndromes as well as Munchausen syndrome by proxy are variants of factitious disorders. They are challenging conditions in Medicine despite the current technology and knowledge on mind-body boundaries.
